# *BRAF* Alteration in Central and Peripheral Nervous System Tumors

**DOI:** 10.3389/fonc.2020.574974

**Published:** 2020-09-15

**Authors:** Komal Srinivasa, Kevin A. Cross, Sonika Dahiya

**Affiliations:** ^1^Department of Pathology & Immunology, Washington University School of Medicine, St. Louis, MO, United States; ^2^Department of Neurosurgery, Washington University School of Medicine, St. Louis, MO, United States

**Keywords:** *BRAF*, CNS, PNS, tumor, diagnosis, prognosis, targeted-therapy

## Abstract

*BRAF* (alternately referred to as v-raf murine sarcoma viral oncogene homolog B1) is a proto-oncogene involved in the mitogen-activated protein kinase (*MAPK*) pathway. *BRAF* alterations are most commonly missense mutations or aberrant fusions. These mutations are observed in numerous primary central nervous system tumors as well as metastases. This review discusses the prevalence of *BRAF* alteration within select notable CNS tumors, and their prognostic associations. Included are some novel entities such as diffuse leptomeningeal glioneuronal tumor (DLGNT), polymorphous low grade neuroepithelial tumor of the young (PLNTY), and multinodular and vacuolating neuronal tumor (MVNT). Knowledge of this gene’s integrity in CNS and PNS tumors can have profound diagnostic and therapeutic implications. Also reviewed are the current state of targeted therapy against aberrant *BRAF* as it pertains mostly to the CNS and to a lesser extent in PNS, and certain diagnostic aspects.

## Introduction

Personalized medicine has revolutionized cancer care in the 21st century, particularly in the areas of diagnosis and treatment. The application of this knowledge to CNS cancer has been variable. While the incorporation of molecular findings has become routine in diagnosing CNS tumors, particularly with the revised 2016 WHO guidelines, targeted therapies have not become similarly common. Patients with gliomas and other primary CNS tumors have a pressing need for new therapies. Five-year survival for primary central nervous system tumors is an estimated 59–64% (1).

In this review, we focus on the proto-oncogene *BRAF* and its relevance in these aspects of CNS and PNS tumors. We detail its normal and pathologic function, its prevalence and prognostic relevance in select primary and metastatic neoplasms, and finally challenges and future directions of targeted therapy.

### BRAF Function

**FIGURE 1:**
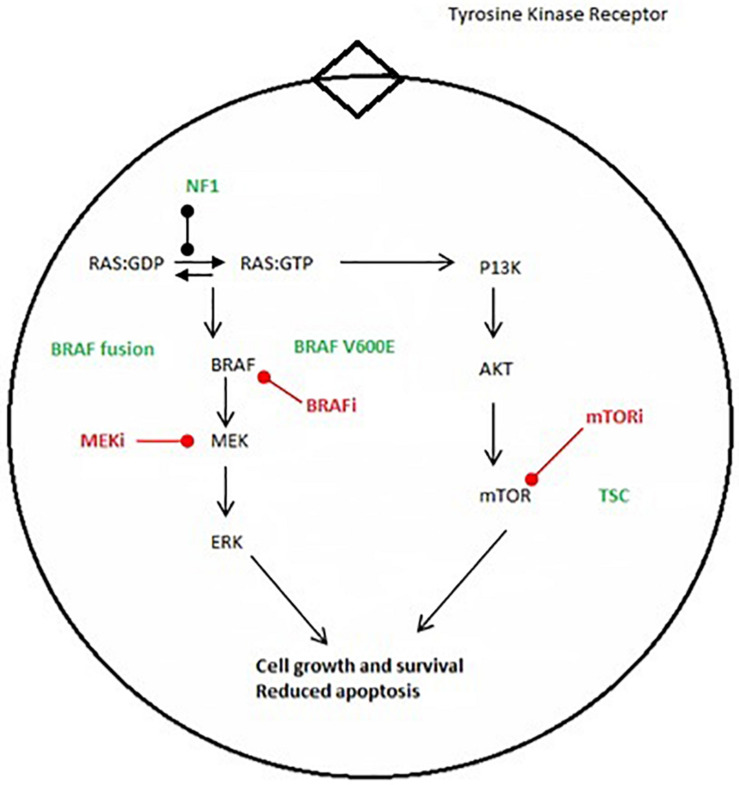
Major oncogenic pathways MAPK and mTOR pathways. Green font shows major genetic alteration. Red font shows proposed molecular therapy.

*BRAF*, alternately referred to as v-Raf murine sarcoma viral oncogene homolog B1, encodes for one of three members of the rapidly accelerating fibrosarcoma (RAF) serine/threonine kinase family. It is located on the long arm of chromosome 7 at position 34. It normally functions as part of the mitogen activated protein kinase (MAPK) pathway, enabling cells to respond to extracellular growth signals. Classically, these growth signals are recognized by tyrosine kinase receptors residing within the plasma membrane, leading to a cascade of phosphorylation events involving protein kinases of the Ras, Raf, MEK, and MAPK/ERK families. MAP kinases, in turn, activate various signals promoting cell growth and survival (Figure 1).

Owing to this role, the MAPK pathway is frequently implicated in human cancer. Derangement can occur by activating events in its upstream signals, or by loss of function in regulatory proteins, such as neurofibromin-1. Gain-of-function mutations in *BRAF* commonly result from pathologic fusion or missense mutations, these occurring in near mutually exclusive fashion (2). In addition to its promotion of cell growth and survival, oncogenic *BRAF* is also known to induce cell senescence (3).

### Mechanisms of Pathologic Activation

Gain of function in *BRAF* results primarily from aberration in the N-terminal portion of the protein, which encodes for an auto-inhibitory domain. Without this domain the protein’s function is uncoupled from upstream signals and remains constitutively active. *BRAF* fusion proteins frequently lack this auto-inhibitory domain entirely. Fusions typically arise from tandem duplications (70% of cases), and less often from deletions and insertions (<3% cases) (4, 5). In the commonest variant, the 5′ segment of the *KIAA1549* gene replaces that of *BRAF* (6). Numerous *KIAA1549:BRAF* fusion variants have been described involving different exons (Table 1). However, 80% of fusions involve exons 15 or 16 of *KIAA1549* with exon 9 of *BRAF* (6).

**TABLE 1:** Patterns *of KIAA1549:BRAF fusion* (32, 103, 104).

**Fusion-Involved Exons *KIAA1549: BRAF***	**Prevalence (%)**
16:9	68%
16:11	10%
15:9	9%
18:10	3%
19:7	3%
15:11–18	3%
17:10–18	3%

Single nucleotide mutations can lead to similar effects (6). The most frequent and well-studied missense mutation substitutes valine for glutamate at position 600 (*V600E*) (7). Other, less frequent, variants include *V600D, V600R, and V600K* (8). These mutations are thought to induce gain of function by mimicking the phosphorylated state of the protein, as they appear in close proximity to regulatory phosphorylation sites at codons 598 and 601.

We detail below how distinguishing between these two mechanisms may impact patient care. And, although rare, it should be noted that *BRAF* fusions and single nucleotide mutation may occur concurrently, as is the case in an estimated 1–3% of low grade gliomas, 3% of PA and PXA, and 1.6% of pilocytic astrocytomas (5, 9) (Table 2).

**TABLE 2:** The frequency *BRAF* aberrations in primary and metastatic tumors to the CNS (PA, pilocytic astrocytoma; PXA, pleomorphic xanthoastrocytoma; DA, diffuse astrocytoma; GBM, glioblastoma; GG/GC, ganglioglioma/gangliocytoma; DIA, desmoplastic infantile astrocytoma/ganglioglioma; DNET, dysembryoplastic neuroepithelial tumor; SEGA, subependymal giant cell astrocytoma; DLGNT, diffuse leptomeningeal glioneuronal tumor; LCH, Langerhans cell histiocytosis; MPNST, malignant peripheral nerve sheath tumor).

**Tumor type**	**Frequency of *BRAF* aberration**
**Primary CNS tumors**	
**Glial**	
PA	*KIAA1549:BRAF* fusion: 50–85% *BRAF* single nucleotide variant: 9–15%
PXA	*BRAF* single nucleotide variant: 63–70%
PXA with anaplasia	*BRAF* single nucleotide variant: 38%
DA	*KIAA1549:BRAF* fusion: 8% *BRAF* single nucleotide variant: up to 14%
Anaplastic astrocytoma	*BRAF* single nucleotide variant: 15%
Oligodendroglioma	*BRAF* single nucleotide variant: 3%
Anaplastic oligodendroglioma	0%
GBM	*BRAF* single nucleotide variant: 9%*
Ependymoma	0%
**Glioneuronal**	
GG/GC	*KIAA1549:BRAF* fusion: 25% *BRAF* single nucleotide variant: 13–56%
DIA/G	*BRAF* single nucleotide variant: 11%
DNET	*BRAF* single nucleotide variant: 51%
SEGA	*BRAF* single nucleotide variant: 43%
DLGNT	*KIAA1549:BRAF* fusion: 65%
**Meningioma**	
Meningioma	*BRAF* single nucleotide variant: 0–3%**
**Embryonal and neuronal tumors**	
Medulloblastoma	0%
CNS primitive neuroectodermal tumor	0%
Atypical teratoid/rhabdoid tumor	0%
Central neurocytoma	0%
**Other tumors**	
Papillary craniopharyngioma	*BRAF* single nucleotide variant: 96%
Adamantinomatous craniopharyngioma	0%
LCH	*BRAF* single nucleotide variant: 50%
Glomus tumor	*BRAF* single nucleotide variant: 6%
Hemangioblastoma	0%
Pituitary adenoma	0%
**Peripheral nervous tumors**	
MPNST	*BRAF* single nucleotide variant: 20%
Schwannoma	0%
Neurofibroma	0%
**Metastatic tumors**	
Melanoma	*BRAF* single nucleotide variant: 41–60%
Papillary thyroid carcinoma	*BRAF* single nucleotide variant: 56%
Colorectal carcinoma	*BRAF* mutation: 10–18%

*Seen with epithelioid and giant cell morphology and in the pediatric population ** rhabdoid subtype (7, 8, 32).

## Primary CNS Tumors

### Gliomas

#### Low Grade Glioma

##### Pilocytic astrocytoma

**FIGURE 2:**
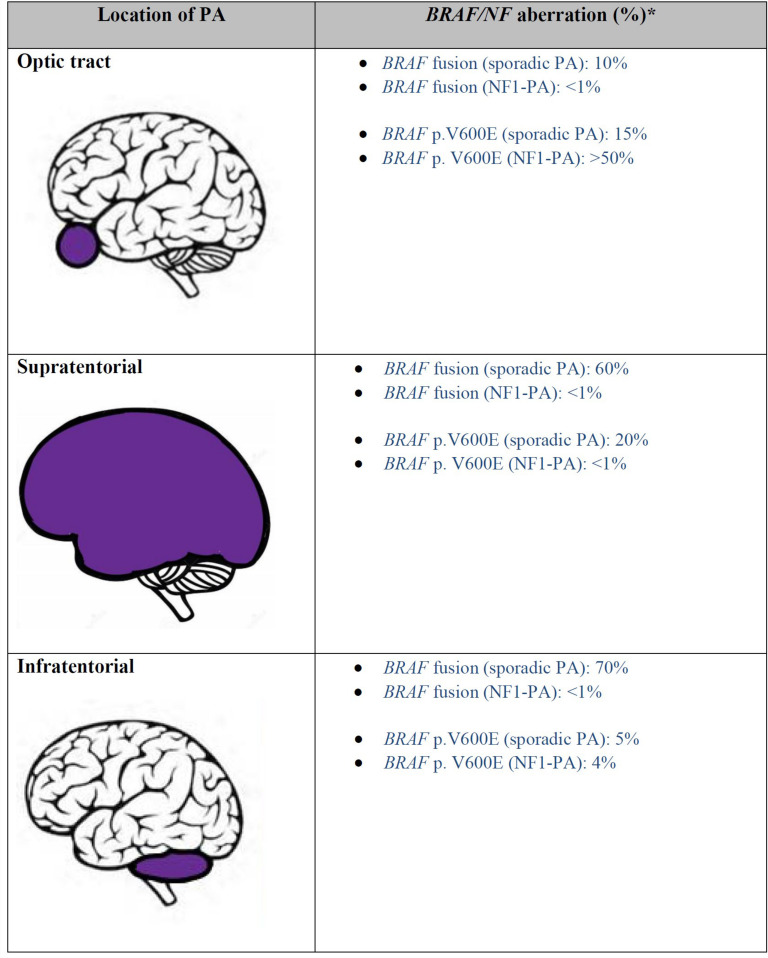
Frequency of *BRAF* alteration in pilocytic astrocytoma based on location (References: 5, 7, 8, 18, 19, 102). *: all the listed values are approximate numbers. NF1, Neurofibromatosis 1; PA, pilocytic astrocytoma.

Pilocytic astrocytoma (PA) is the commonest primary childhood CNS tumor, accounting for 30% of the total. These tumors are illustrative for the role of *BRAF* testing. Over two thirds of these tumors arise within the posterior fossa (10). Roughly 85% of cases are sporadic, and 15% are associated with the inherited tumor predisposition syndrome neurofibromatosis type 1 (NF1) (11) (Figure 2).

Despite histologic similarity, sporadic and NF1 associated pilocytic astrocytomas exhibit different characteristics in terms of location and clinical course. While the vast majority of pilocytic astrocytomas are sporadic and arise in the posterior fossa, in NF1 patients 50–76% occur in the optic pathway and are known as optic pathway gliomas (11). NF-1 patients also frequently develop PA in the cerebral hemispheres. In fact, just 4% of cerebellar PAs are associated with NF1. Nevertheless, both supratentorial and infratentorial locations of PA frequently require treatment. Optic pathway gliomas, which may threaten vision, are most often treated with chemotherapy, while posterior-fossa PAs, which threaten brainstem compression or obstructive hydrocephalus, are most often resected.

**FIGURE 3:**
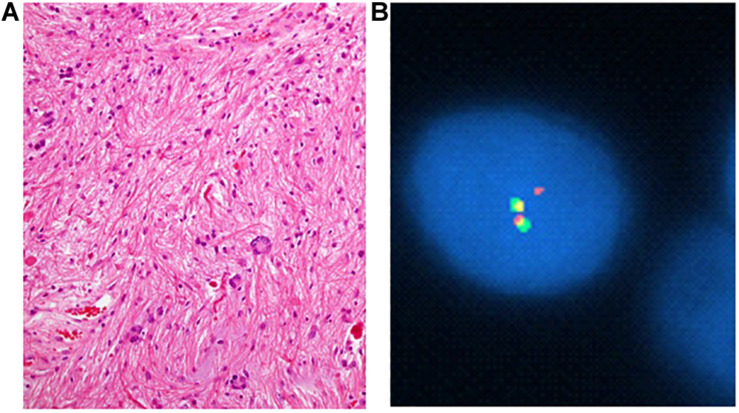
Pilocytic astrocytoma with compact areas harboring piloid cells, abundant Rosenthal material, and some multinucleate cells (“pennies on a plate”) (H&E; **A**) and florescence *in situ* probe showing *KIAA1549:BRAF* fusion. Yellow signal signifies fusion **(B)**.

These differences are believed to result from differing underlying oncogenic mechanisms, namely *BRAF* and *NF1* (−/−) activation of the MAPK and PI3K/mTOR pathways. Sporadic PAs more commonly demonstrate *BRAF* fusion rather than single nucleotide mutations, as nearly 80% of cerebellar PAs harbor fusions, which most often involve *KIAA1549* (Table 1 and Figure 3). However, in fewer than 5 percent of cases the fusion involves other genes involved in the MAPK pathway such as *FAM131B, SRGAP, QK1, RNF130, CLCN6, MKRN1, GNA11, FZR1, and MACF1* (2, 4, 5, 12–14). *V600E* is not thought to play a major role in their pathogenesis. Its prevalence in all pediatric PAs regardless of location is estimated to be around 6% (15).

The signature molecular aberration in NF1-associated PAs, by contrast, is loss of neurofibromin. This product of the *NF1* gene is a negative regulator of Ras. Its loss activates both the MAPK and PI3K/mTOR signaling pathways (11, 16).

While cases have been reported of optic gliomas bearing both *KIAA1549:BRAF* and *NF1* loss, these are rare (17). Therefore, these markers define two seemingly mutually exclusive subgroups of this entity.

*BRAF* genotype may also have prognostic implications. Some have argued *KIAA1549:BRAF* may lead to a less-aggressive phenotype. In a study of 58 subtotally resected PAs, Hawkins et al. found 5-year progression-free survival to be 65% in fusion patients and 17% in wild type (*p* = 0.002) (18). While Horbinski et al. found *BRAF* fusion in PAs to be associated with better prognosis as well, this was not statistically significant (19). It is speculated that the less-aggressive phenotype in *BRAF* fusion patients may result from the phenomenon of oncogene-induced cell senescence (18).

Overall, survival rates in pediatric pilocytic astrocytoma are excellent, generally exceeding 90% at 5 years (20, 21).

##### Diffuse glioma

The diagnosis of diffuse glioma has evolved with the incorporation of molecular markers, but remains particularly challenging. The 2016 WHO revised guidelines identified the importance of *IDH1/2* and 1p/19q codeletion status for classification of diffuse gliomas into astrocytic or oligodendroglial phenotypes. However, pediatric diffuse gliomas rarely demonstrate these findings (22). *BRAF* aberrations, by contrast, are more frequent in the pediatric population (6, 22). While studies in adults estimate the prevalence of all *BRAF* mutation at less than 1% (7, 23), in children and adolescents rates are estimated to be 3% for fusion, and 8–43% for *V600E*. (6, 15, 24). This might indicate somewhat distinct molecular underpinnings in evolution of diffuse gliomas in adults when compared with the pediatric population.

There is a low incidence of diffuse glioma in children, with less than 10% of pediatric low grade gliomas falling into this category in some studies (20). In addition, they are frequently examined in combination with other subtypes of low-grade glioma in studies. Prognostic predictions in this population are therefore challenging. However, limited data do suggest that *V600E* may be associated with more aggressive phenotype. In a large series of pediatric low-grade gliomas, Lassaletta et al. revealed *V600E* mutations in 10 of 23 diffuse astrocytomas (43%), 2 of 15 pilomyxoid astrocytomas (13%), and 14 of 70 low grade gliomas not otherwise specified (20%) (24). They analyzed outcomes from these patients in combination with other low grade gliomas and found *V600E* was associated with worse 5 year (50.1 vs. 72%) and 10 year PFS (27 vs. 60.2%) (24). The cIMPACT-NOW update four guidelines suggest using a designation “diffuse glioma, *BRAF* V600E-mutant” for tumors exhibiting a *BRAF V600E* mutation (25).

In adults, grade II diffuse gliomas account for 24% all CNS cases and are found largely within the cerebral hemispheres. *BRAF* mutations occur in less than 1%, limiting prognostic assessments. The most important predictors of prognosis in this tumor subset remain extent of resection, tumor location, and patient age (6, 19).

##### Pleomorphic xanthoastrocytoma

Pleomorphic xanthoastrocytoma (PXA) accounts for less than 1% of all astrocytic tumors, but is more frequent in children (10, 26). These tumors show a predilection for the temporal lobe and superficial cortices (10). *BRAF V600E* is highly enriched in this population, with 2/3 of adult and pediatric cases bearing it, including a smaller number of cases with anaplasia (33% of such cases) (7).

Overall, prognosis for PXA is favorable, with recurrence-free survival estimated to be 64% following initial resection (27). The prognostic utility of *BRAF V600E* has been investigated, but remains indeterminate (27–29).

In addition to *BRAF*, PXA frequently features aberrations in *CDKN2A*, which encodes for the cyclin dependent kinase inhibitor p16 (9). In a study of 38 primary and anaplastic PXAs, 87% were found to exhibit a homozygous deletion. However, this deletion did not correlate with *BRAF* status, as it was distributed equally among wild type and mutant tumors (30). In this same cohort, *BRAF* status did not clearly confer survival benefit.

#### High Grade Gliomas

**FIGURE 4:**
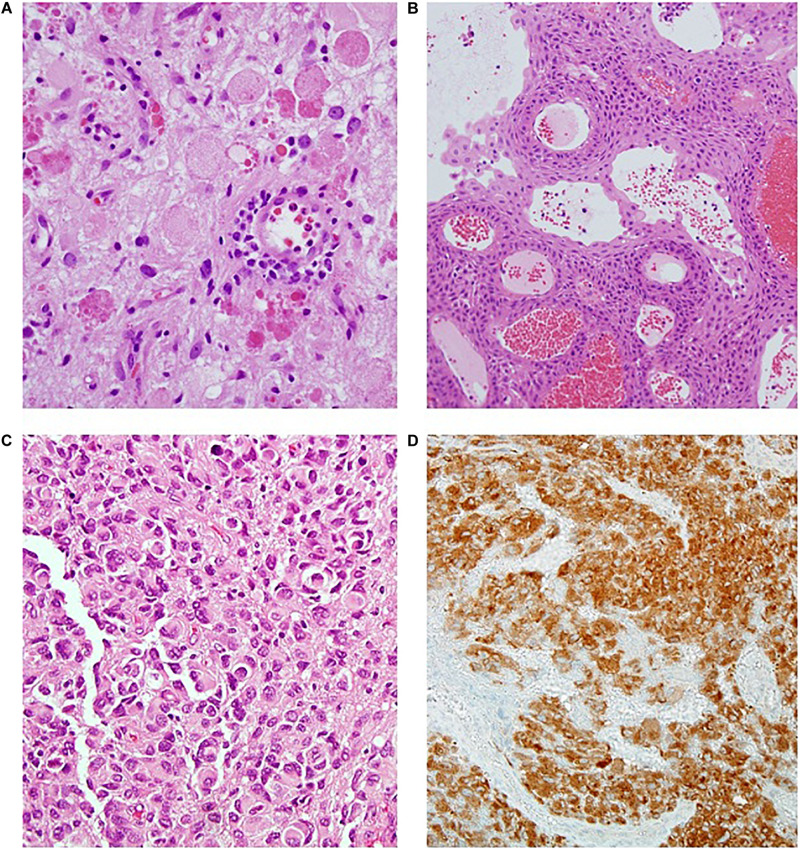
**(A)** Ganglioghoma (H&E); **(B)** Papillary craniopharyngioma (H&E); **(C)** Epithelioid glioblastoma (H&E); **(D)** BRAF immunostaining showing diffuse positive staining (BRAF IPX).

The frequency of BRAF mutations in adult GBM is estimated to be 1–3% (31). However, certain cohorts have higher frequencies. Glioblastomas in teenage and young adults are enriched for the *V600E* mutation, and it may be observed in up to 50% of the epithelioid variant (31, 32, 33) (Figure 4C).

### Glioneuronal Tumors

#### Ganglioglioma (GG)

Ganglioglioma accounts for 7% of childhood and adolescent primary CNS tumors (26). These are slow growing, circumscribed tumors that may harbor both cystic and solid components (10). Histologically they show neuronal (comprised by generally ganglionic) and glial elements in variable proportions (Figure 4A). *BRAF* missense mutations are estimated to be found in half of specimens, with *BRAF* fusions seen in another 10–25% (6, 7, 34). In a series of 53 pediatric gangliogliomas, Dahiya et al. found *BRAF V600E* to be associated with shorter recurrence free survival (35).

#### Diffuse Leptomeningeal Glioneuronal Tumor

Diffuse leptomeningeal glioneuronal tumor (DLGNT) is a rare entity added only recently to the WHO classification of CNS tumors, and these have not been assigned a WHO grade as their natural history is not clearly delineated yet (10). It is characterized by leptomeningeal spread of histologically monomorphic, oligodendroglial-like cells, usually in children and adolescents. Molecularly, it features loss of 1p, without an abnormality in IDH (10, 36, 37). In a review of 30 cases, Deng et al. demonstrated pathologic activation of the MAPK pathway in 80%, and suggested this to be a hallmark molecular feature of this tumor (37). In 66% of their specimens, this was a result of *KIAA1549:BRAF* (37). They further defined and characterized two molecular subclasses using DNA methylation profiling. Class 1 was found more commonly in younger patients (median age 5), with frequent 1p/19q codeletions (47%), and was associated with 100% 5-year progression free survival. Subclass 2 tended to arise in older patients (median age 14), who experienced 43% 5 year overall survival. *BRAF* fusions were observed equally in the two groups (76% in class 1 vs. 77% in class 2).

#### Polymorphous Low Grade Neuroepithelial Tumor of the Young (PLNTY)

In 2017, Huse et al. described a new entity of low-grade, oligodendroglioma-like neuroepithelial tumor, naming this polymorphous low grade neuroepithelial tumor of the young (PLNTY) (38). These are, generally, epileptogenic tumors located in the subcortical temporal lobe and associated with dystrophic calcifications (39, 40). Histologically, these tumors show a diverse astrocytic and ependymal appearance, though frequently there is an oligodendroglioma-like component (38). There is immunoreactivity for CD34 and OLIG2. Tumor cells lack *IDH1* R132H or 1p/19q co-deletion. Nearly all, however, featured overactivation of the MAPK pathway (38). In Huse’s original series, 3 of 7 cases were *BRAF V600E* mutant and the remaining cases exhibited *FGFR2/3* fusion events (no *BRAF* fusion was detected) (38).

#### Multinodular and Vacuolating Neuronal Tumor (MVNT)

Multinodular and vacuolating neuronal tumor (MVNT) is a provisional entity included in the 2016 WHO classification of CNS tumors (10). Due to their indolent behavior and ease of surgical excision, Huse et al. suggest assigning this entity a WHO grade I designation, however, a formal WHO grade has not yet been assigned (41). MVNT is a supratentorial tumor which generally appears on MRI as multiple subcortical, FLAIR-hyperintense nodules (42). Histologically the tumor cells arrange into clusters and have conspicuous intracytoplasmic and stromal vacuolation (41, 43). They express OLIG2, CD34, and are also labeled by anti-HuC/HuD with variable expression of synaptophysin, and no reactivity for chromogranin, NeuN and/or neurofilament (10, 41). In their original description Pekmezci et al., analyzed eight samples using phospho-ERK immunohistochemistry and demonstrated uniform MAPK pathway overactivation in this tumor (43). With next-generation sequencing, they subsequently revealed mutations in *BRAF*, *MAP2K1*, and *FGFR2* as candidate drivers (43). Although the sample size in this study was small, interestingly, the two *BRAF* mutations observed were *L597R* and *G469S* and not *V600E*. Though uncommon, these have previously been described in langerhans cell histiocytosis, Erdheim-Chester disease, and melanoma (43).

### Non-neuroepithelial Tumors

#### Langerhans Cell Histiocytosis

Langerhans cell histiocytosis (LCH) is the most common histiocytic tumor affecting children. Within the CNS, these are usually found in the skull or hypothalamus. *BRAF* point mutation is found in an estimated 69% of cases, with 47% of those representing *V600E* (44). Other mutations include *T599A, 600DLAT* insertion and *BRAF V600D* (45).

*BRAF* alteration portends worse clinical course, as shown by Heritier et al. where they studied 315 patients, of which 55% were harboring *V600E*, and found this was associated with a seven-fold increased risk of resistance to standard treatment (21.9 vs. 3.3%) (46). These patients also reactivated at a higher rate over a 5 year follow-up (42.8 vs. 28.1%), and experienced more permanent and severe sequelae (46).

#### Craniopharyngioma

Craniopharyngioma is a benign, WHO grade I tumor that has relatively low risk of mortality, but causes significant morbidity in terms of endocrine and visual function (Figure 4B). Interestingly, though the two subtypes (i.e., adamantinomatous and papillary) are associated with strongly dichotomous molecular features, they behave largely similarly in patients, aside from age of initial presentation. In terms of morbidity and mortality, there are to our knowledge no studies correlating tumor subtype with long term outcome. Karavitaki, for example, reviewed 121 cases of craniopharyngioma and found no difference in overall survival at 10 years (47).

In 2014 Brastianos et al. revealed the *V600E* substitution in 95% of 39 papillary specimens (48). Meanwhile, 96% of adamantinomatous subtypes were shown to harbor mutations in *CTNNB1* and all adamantinomatous specimens were *BRAF* wild-type. The authors therefore proposed these markers defined two distinct and mutually exclusive clonal patterns (48). Since the original account, rare adamantinomatous specimens have been discovered to bear *BRAF V600E* (49). However, the specificity of distinct alterations overall remains very high.

#### Glomus Tumor

Glomus tumors are seldomly encountered mesenchymal neoplasms, and typically follow a benign clinical course (50, 51). Most glomus tumors are superficial, less than 2 cm, and surgically curable (52). *BRAF V600E* appears with a frequency of 6–11% (50, 51, 53). Its presence in glomus tumors may predict a more aggressive phenotype (53). Karamzadeh et al. reviewed 102 cases, largely originating from the extremities (53). In this series *BRAF V600E* was not observed in any of 57 benign cases, but was enriched in tumors of uncertain malignant potential (3/14, 21%) and malignant varieties (3/24, 12%). These tumors showed atypical histological features including deep location, size more than 2 cm, infiltrative growth, and mitotic count of ≥5/50 HPFs (53).

## Malignant Peripheral Nerve Sheath Tumors

Malignant peripheral nerve sheath tumors (MPNST) comprise 5–10% of all soft tissue sarcomas, are usually deep seated, and arise from a peripheral nerve trunk (54). 50% are associated with NF1. Overall, NF1 patients have an 8–13% lifetime risk of developing it (55). The rate of *BRAF V600E* in all MPNSTs is estimated to be 6.5% (2.9% in NF1, 11.9% sporadic) (56). *BRAF* fusions have also been reported, though these are less frequent. (56) Little is currently known about the prognosis of a *BRAF* mutation in MPNSTs (55, 56), though one group found no statistically significant differences in survival or time to between mutant and wild-type cohorts (55).

## Metastatic Tumors to the CNS

Brain metastases are the most common intracranial neoplasm in adults, with an incidence estimated at between 8.3 and 11 per 100,000 (57). The most frequent primary tumors are lung, melanoma, breast, renal, and colorectal (57). The role of *BRAF* is under intense study in several of these contexts. CNS-specific outcomes, however, are ill-defined.

Estimates of *BRAF* aberration in non-small-cell lung carcinoma are less than 10% (58). In one large series of 1,046 cases, rates were 4.9% in adenocarcinoma and 0.3% in squamous cell carcinoma (58). *V600E* accounted for 57% of the total aberrant *BRAF* in cases of adenocarcinoma and squamous cell carcinoma in this study, and was independently associated with poorer prognosis. The study did not examine CNS specific outcomes, however.

In melanoma, *BRAF* mutations are found in approximately 50% of stage IV disease, and of those roughly 70% are *V600E* and 20% are *V600K* (59). *BRAF* mutation is associated with slightly younger age at primary diagnosis, but does not appear to influence overall survival (60, 61). Kotecha et al. found that for patients who had stereotactic radiosurgery, the 12 month local failure rate was higher amongst patients with *BRAF* wildtype metastatic melanoma compared with *BRAF* mutant cases (22% compared with 6% (62). Prevalence of *BRAF* mutation in brain-specific metastases is similar to prevalence in other metastatic sites (63).

*BRAF* mutations are rare in breast and renal carcinoma, but they do occur in 5–10% of patients with metastatic colon carcinoma (64). Over 95% of these are *V600E* (64). These patients exhibit shorter overall survival as compared to their wild type counterparts (65). CNS-specific outcomes are unfortunately lacking.

Approximately 25–83% of papillary thyroid carcinoma harbor *BRAF* mutation, where it is associated with a more aggressive phenotype (66). However, this tumor rarely metastasizes to the brain and in one meta-analysis, *BRAF* status was not related to distant metastasis (66).

## Successes and Challenges in Targeting *BRAF*

To date, oncogenic *BRAF* has been targeted with varying success scale in humans in melanoma, non-small cell lung cancer, colorectal carcinoma, and thyroid carcinoma, as well as gliomas and glioneuronal tumors. By far, the majority of clinical experience has been gained in metastatic melanoma. This experience has highlighted several challenges that will inform treatment of patients with *BRAF*-associated CNS or PNS disease. Additionally, if these treatments are to gain clinical relevance, CNS-specific obstacles must be considered.

The first *BRAF* inhibitors (BRAFi) described were non-specific “multikinase” inhibitors. These were developed before recognition of widespread *BRAF* mutation, including *V600E*, in human cancer. Sorafenib (Bayer/Onyx) is the archetypal agent of this family. This drug was FDA approved for use in advanced renal cell carcinoma in 2005, for hepatocellular carcinoma in 2007, and for metastatic thyroid carcinoma in 2013. In a phase III trial in metastatic melanoma, the addition of sorafenib to carboplatin/paclitaxel failed to improve overall survival (67). This was likely a result of poor efficacy against *V600E*. Importantly, sorafenib was also the first BRAFi to be associated with paradoxical MAPK activation in *BRAF* wild-type and *BRAF* fusion cells. This was reported *in vitro* in 1999 (68), but its clinical significance was recognized only later, with the observation that patients treated for renal cell carcinoma developed keratoacanthomas and squamous cell carcinomas (69). In 2011 this adverse effect was highlighted in a phase II trial of children with low-grade astrocytoma, during which 9 of 11 patients unexpectedly and rapidly progressed following three cycles of treatment with the drug (70). This led to a premature termination of the trial. Subsequent experience has reinforced opinion that this unexpected result was due to paradoxical ERK activation (70, 71).

Second generation inhibitors include vemurafenib, dabrafenib, and encorafenib, all of which are currently FDA approved for use in metastatic melanoma. Unlike sorafenib, these were specifically engineered to target *V600E*-bearing *BRAF*. Vemurafenib (PLX4032, Zelboraf, Plexxikon/Genentech) was developed in 2008 by a structure-guided approach and rapidly underwent phase I and II trials (72). In 2011 data from a phase III randomized controlled trial comparing vemurafenib to dacarbazine in patients with unresectable stage IIIc or IV disease were reported (73). The vemurafenib cohort experienced 63% relative risk reduction in death and 74% relative risk reduction in tumor progression (71). Results of a trial comparing dabrafenib (GSK2118436, Tafinlar, GlaxoSmithKline) to dacarbazine were similarly positive (74). Encorafenib was granted approval in 2018 for use in combination with binimetinib, a MEKi.

Second-generation inhibitors also induce MAPK activation in non-V600E cells, leading to the adverse effect of secondary cutaneous neoplasm in 14–26% of patients (75). Similarly to sorafenib, this includes cells bearing *BRAF* fusion protein. *In vitro*, cortical neurospheres containing *KIAA1549-BRAF* treated with an analog of vemurafenib exhibit paradoxical growth (71).

While second generation inhibitors target *V600E*, an FDA-approved therapy specific to *KIAA1549:BRAF* is lacking. Current strategy for these tumors is toward targeting other molecules in the MAPK pathway. *In vitro*, cells bearing *BRAF* fusion are susceptible to MEK inhibitors such as selumetinib, binimetinib, trametinib, and cobimetinib (76, 77). *In vivo*, success has also been achieved in some patients. One phase II trial studied selumetinib in recurrent or refractory *BRAF*-aberrant or NF1 associated low-grade gliomas (78). In a subset of 18 patients harboring the *KIAA1549:BRAF* fusion, seven (39%) exhibited partial response (78). Two randomized, controlled trials are currently comparing selumetinib with standard chemotherapy in pediatric low-grade glioma (78). In addition, next-generation Raf inhibitors in phase I/II trials may also have efficacy against *BRAF* fusion proteins (79, 80).

Acquired resistance has proved to be the major obstacle to durable response in patients with a variety of cancers treated with BRAFi. In melanoma, median response duration to BRAFi monotherapy is just 5–8 months (81). It is generally accepted that acquired resistance to BRAFi occurs via reactivation of the MAPK and, to a lesser extent, PI3K/mTOR pathways. This can result, for example, from overexpression or mutation in Ras that bypasses Raf, or by secondary activating mutations in MEK (82, 83). Combination BRAFi + MEKi has improved clinical responses, likely by addressing this escape mechanism. In melanoma, BRAFi + MEKi extends duration of response to 9.5 months (81). Consequently, combination therapy is now standard-of-care. Resistance to combination therapy is being investigated, but appears to involve similar mechanisms of upstream activation and parallel activation of the PI3K/mTOR pathway.

One CNS-specific obstacle to *BRAF* targeted therapy may be the blood-brain or blood-tumor barrier. There is some suggestion of this effect in data from trials in metastatic melanoma. The phase II COMBI-MB trial evaluated the efficacy of dabrafenib plus trametinib in patients with *BRAF V600E* positive melanoma with asymptomatic brain metastases (84). Whereas median response duration was 10.2 months for extracranial disease, it was just 6.5 month for intracranial disease (84). Some preclinical data do suggest that brain bioavailability of BRAFi may be limited by this barrier. *In vitro*, Durmus et al. found vemurafenib was efficiently effluxed by transporters of the ATP-binding cassette family (ABC), which are active in the blood-brain barrier (85). *In vivo*, those mice coadministered vemurafenib with elacridar, an inhibitor of *ABCG2*, experienced increased brain concentrations of drug (85).

In glioma, clinical trial data of BRAFi are thus far limited. VE-BASKET was a non-randomized, open-label study that evaluated vemurafenib monotherapy in patients with WHO grades I-IV glioma harboring the *V600E* mutation (86). It enrolled 24 subjects: 7 PXA, 2 PA, and 15 grade III/IV gliomas. 20% of these patients had partial response, 40% had stable disease and one complete response was observed in a PXA. Overall, responses in lower-grade tumors were better, though sample sizes were small and this difference did not reach statistical significance (86). Hargrave et al. more recently reported the results of dabrafenib monotherapy in a subset of pediatric patients with recurrent, *BRAF* mutant, low grade glioma (87). Of their 32 patients, 44% experienced objective response by response assessment in neuro-oncology (RANO) criteria, as compared to the apparent rate of 10% in historical controls treated with standard of care. Mean duration of response was 11 months. Other case series and reports have confirmed similar findings (31, 88–90).

There is reason to believe BRAFi + MEKi may be superior to BRAFi monotherapy in glioma as in other cancers. In an animal model of *V600E* high-grade glioma, Grossaeur et al. documented longer treatment effects when analyzing the frequency of proliferative tumor cells with combination therapy (91). They also suggested combined BRAFi + MEKi therapy may prevent secondary RAS driven cancers, such as squamous cell carcinomas, which can be seen with monotherapy (91). Small series of combination in both pediatric and adult high grade glioma have also demonstrated occasional rapid and durable responses (31, 92). A phase II trial (NCT02684058) is currently underway studying dabrafenib and trametinib in children and adolescent patients with *BRAF V600E* low grade glioma and adults with relapsed or refractory high grade glioma (92).

Papillary subtype craniopharyngioma is another obvious target for investigation given its high (95%) prevalence of *V600E*. Several groups have described cases of dramatic and sometimes-sustained response to BRAFi with or without MEKi (93–98). A multi-institutional phase II trial (Alliance A071601) is currently underway to test the combination of vemurafenib and cobimetinib) (98).

## *BRAF* Testing in Neuropathology

In testing for *BRAF* aberration, knowledge of the broader clinical context, as well as the limitations of tissue and available tests, will help to optimize testing algorithms.

Molecular platforms are the “gold standard” of *BRAF* analysis. These include Sanger Sequencing, allele specific polymerase chain reaction (PCR), pyrosequencing, High Resolution Melting curve and Next Generation Sequencing (NGS) (99, 100). High fidelity sequencing is the most sensitive test for a wide variety of possible aberrations. However, the ability to perform these tests may be limited by the quality and quantity of tissue and the ability to extract high-quality DNA. These tests may also be cost-prohibitive.

Such circumstances thus call for immunohistochemistry as a triage before performing the molecular testing. It has been shown that the most frequent and clinically relevant point mutation is *V600E*, against which a commercially available monoclonal antibody currently exists (Figure 4D). Easy to perform and relatively inexpensive, this stain may be used either as a screen to be followed by more dedicated testing, or alternately in isolation. The sensitivity of this test has been shown to be near, or at, 100% (101). However, false negatives may still result from freezing or surgical cautery artifact, prompting some caution (2).

Fluorescence *in situ* hybridization fusion and break apart probes for *KIAA1549-BRAF* provide a reliable method for diagnosis of fusion genes (Figure 3B). Because of the heterogeneity of observed *KIAA1549-BRAF* fusions, the tester must be attuned to whether the probe is a fusion or break apart probe, and in the case of a break apart probe, the specific exon (Table 1).

Even with detailed molecular analysis available, the limitations to apply this knowledge must be recognized as our experience is still evolving. An example from the pediatric context is instructive. Given a limited sample of a pediatric brain tumor featuring piloid glial cells with or without Rosenthal fibers, and eosinophilic granular bodies, the presence of a *BRAF* fusion may be highly suggestive, but not pathognomonic, of a pilocytic astrocytoma (36, 102). Molecular findings always require correlation with the patient’s clinical, histological, and radiologic details.

## Conclusion

*BRAF* alterations are identifiable using current diagnostic techniques, and at present play an important role in the pathologic workup of CNS tumors and to a lesser degree in PNS. *BRAF* targeted therapies, particularly newer generation and in combination, hold promise for use against several subtypes of tumors.

## Author Contributions

KS, KC, and SD contributed to writing and editing of this manuscript. SD provided supervision of the writing process. All authors contributed to the article and approved the submitted version.

## Conflict of Interest

The authors declare that the research was conducted in the absence of any commercial or financial relationships that could be construed as a potential conflict of interest.
